# Supplementary material for: Evolving Together: Cassandra Retrotransposons Gradually Mirror Promoter Mutations of the 5S rRNA Genes
Source: Mol Biol Evol. 2024 Jan 23;41(2):msae010. doi: 10.1093/molbev/msae010 (PMC10853983; doi:10.1093/molbev/msae010)

Suppl. figure 2: Cassandra variants within the Asteraceae. Two variants of Cassandra sequences can be identified within the Carduoideae. These sequences share the same internal region but show different LTR sequence information, although each of them harbour conserved signals for transcription by Polymerase II or III: either the promoter boxes of 5S rDNA (light blue) or a TATA box (brown, A). LTR alignments of Asteraceae Cassandras show different LTR sequence informations for Cassandras and the Cassandra-like non autonomous elements (B). But internal regions of these variants are highly similiar, although Cassandra-like retrotransposons show a longer variant (C). As for *Bidens hawaiensis* (Bhaw) we observe a duplication within the internal region, leading to an usually longer variant. Differences from consensus are highlighted in color (A=red, T=green, C=yellow, G=blue). Consensus was calculated with a treshold of 25% (bases match atleast 25% of the sequences). Cassandra sequence names are shortened by species name: Aann = *Artemisia annua*, Cind = *Chrysanthemum indicum*, Gcor = *Glebionis coronaria*, Tcin = T*anacetum cinerariifolium*, Ccan = *Conyza canadensis*, Humb = *Helichrysum umbraculigerum*, Sson = *Smallanthus sonchifolius*, Mmic = *Mikania micrantha*, Sreb = *Stevia rebaudiana*, Bhaw = *Bidens hawaiensis*, Hann = *Helianthus annuus*, Satr = *Scalesia atractyloides*, Pind = *Pluchea indica*, Alap = *Arctium lappa*, Ctin = *Carthamus tinctorius*, Ccar = *Cynara cardunculus*

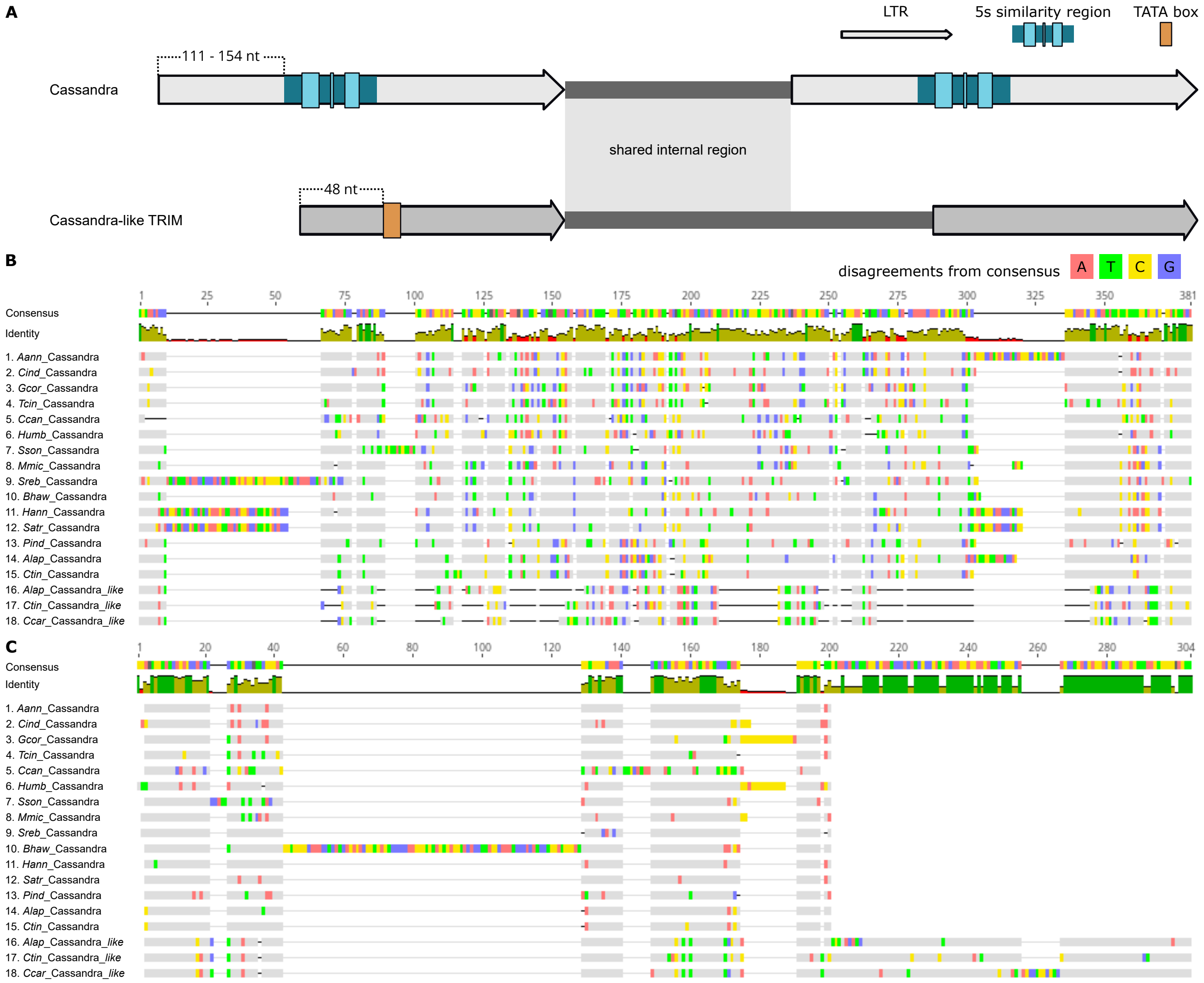

Supplement: msae010_Supplementary_Data [file msae010_supplementary_data.zip › Suppl_figure2_AST_Cassandra_variants.pdf]
