# Supplementary material for: Evolving Together: Cassandra Retrotransposons Gradually Mirror Promoter Mutations of the 5S rRNA Genes
Source: Mol Biol Evol. 2024 Jan 23;41(2):msae010. doi: 10.1093/molbev/msae010 (PMC10853983; doi:10.1093/molbev/msae010)

Suppl. figure 3: Polymorphic motifs in Cassandra LTR - 5S similarity region. Within the conserved 5S region in the Cassandra long terminal repeats (LTRs) we observe variable motifs between the highly conserved promoter boxes (A-Box, Intermediate element - IE, C-Box). Depending on their localization and association with the nearest conserved promoter boxes, they are called MotIE and MotC. For MotIE and MotC we observe shifts in the sequence information (A). To better understand the regions of variability between the Cassandra LTRs, we further investigated the nucleotide compositions in the MotIE and MotC regions and how they map back to the 5S rDNA. Most striking are the differences in nucleotide composition of MotIE and MotC polymorphisms. 45 Cassandra sequences show a MotIE polymorphism with eight observable nucleotide motif variants and only one being species-specific (*J. curcas*). MotC divergence from the 5S rRNA gene was detectable in 37 out of 45 Cassandra sequences Here, variability is even higher and we observe 18 different motifs with nine of them being specific for species of Rosaceae (*M. domestica*), Fabaceae (*G. max*, *L. japonicus*, *P. sativum*) and Asteraceae (*C. canadensis*, *H. umbraculigerum*, *M. micrantha*, *S. atractyloides*, *S. sonchifolius*). Nevertheless, most motif changes appear as stochastic variation and carry no clear phylogenetic signal.
In contrast, there is no observable variability in the corresponding 5S rDNA genes in these regions (B). For 5S rDNA of corresponding genes consensi for mutual and cannonical C-Boxes are shown.

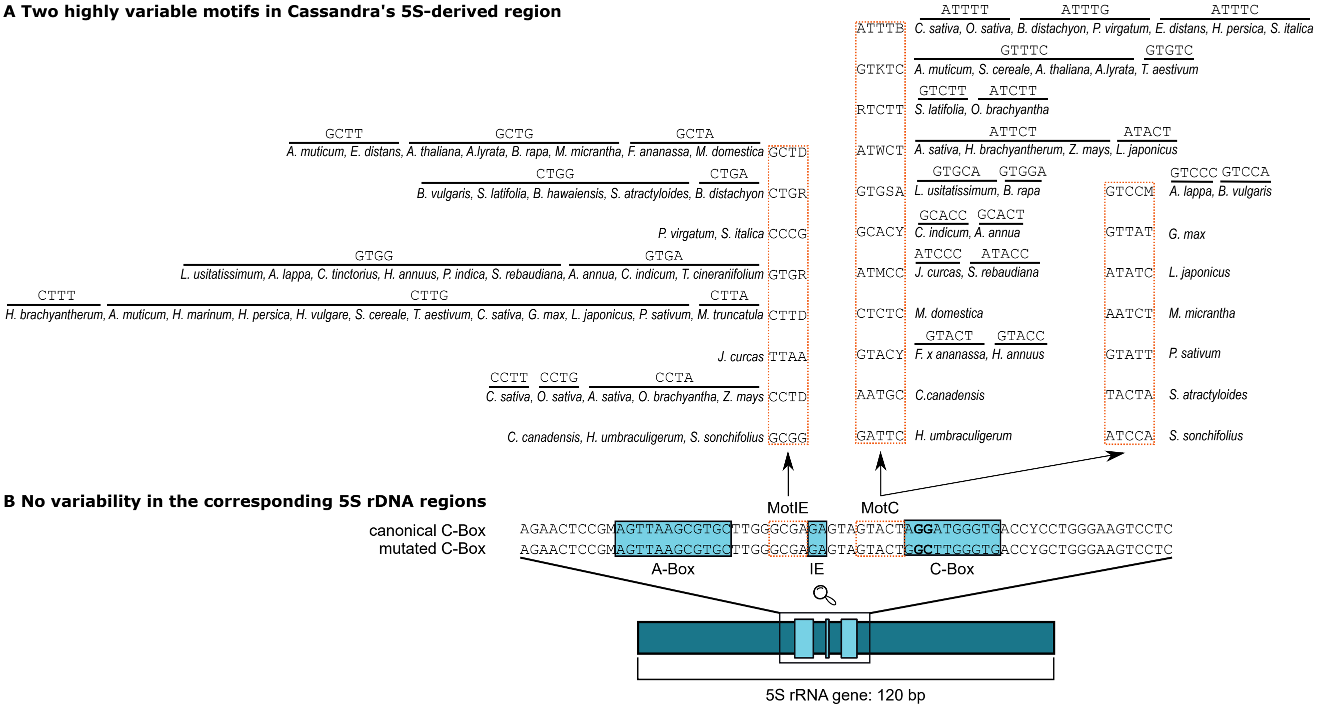

Supplement: msae010_Supplementary_Data [file msae010_supplementary_data.zip › Suppl_figure3_polymorphic_motifs_in_Cassandra_LTR.pdf]
