# Supplementary material for: Evolving Together: Cassandra Retrotransposons Gradually Mirror Promoter Mutations of the 5S rRNA Genes
Source: Mol Biol Evol. 2024 Jan 23;41(2):msae010. doi: 10.1093/molbev/msae010 (PMC10853983; doi:10.1093/molbev/msae010)
Supplement: msae010_Supplementary_Data [file msae010_supplementary_data.zip › Suppl._table1_PLANT_Cassandra_data_mining.pdf]

Suppl. table 1A: Data sources for published Cassandra sequences

plant order	plant family	species	accession number	reference	DOI
Polypodiales	Didymochlaenaceae	*Didymochlaena trunculata*	AY860311.1	Kalendar et al., 2008,	10.1073/pnas.0709698105
Polypodiales	Nephrolepidaceae	*Nephrolepis exaltata*	AY860313.1	Kalendar et al., 2008	10.1073/pnas.0709698105
Cyatheales	Cyatheaceae	*Sphaeropteris cooperi*	AY860310.1	Kalendar et al., 2008	10.1073/pnas.0709698105
Piperales	Aristolochiaceae	*Saruma henryi*	EF125873.1	Kalendar et al., 2008	10.1073/pnas.0709698105
Poales	Poaceae	*Ambylopyrum muticum*	AY603371.1	Kalendar et al., 2008	10.1073/pnas.0709698105
	Poaceae	*Avena sativa*	AY271960.1	Kalendar et al., 2008	10.1073/pnas.0709698105
	Poaceae	*Brachypodium distachyon*	Supplemental material from reference	Gao et al., 2016	10.1186/s13059-015-0867-y
	Poaceae	*Bromus sterilis*	AY271957	Kalendar et al., 2008	10.1073/pnas.0709698105
	Poaceae	*Colpodium drakensbergense*	FJ975775.1	Kalendar et al., 2020	10.3390/ijms21082931
	Poaceae	*Colpodium versicolor*	FJ975776.1	Kalendar et al., 2020	10.3390/ijms21082931
	Poaceae	*Deschampsia antarctica*	EU867815	Kalendar et al., 2020	10.3390/ijms21082931
	Poaceae	*Eremopyrum distans*	AY603372.1	Kalendar et al., 2008	10.1073/pnas.0709698105
	Poaceae	*Henrardia persica*	AY603374.1	Kalendar et al., 2008	10.1073/pnas.0709698105
	Poaceae	*Hordeum brachyantherum*	AY603373.1	Kalendar et al., 2008	10.1073/pnas.0709698105
	Poaceae	*Hordeum marinum*	AY603375.1	Kalendar et al., 2008	10.1073/pnas.0709698105
	Poaceae	*Hordeum vulgare*	AY164585.1	Kalendar et al., 2008	10.1073/pnas.0709698105
	Poaceae	*Oryza brachyantha*	Supplemental material from reference	Gao et al., 2016	10.1186/s13059-015-0867-y
	Poaceae	*Oryza glaberrima*	HM481419.1	Kalendar et al., 2020	10.3390/ijms21082931
	Poaceae	*Oryza minuta*	HM481420.1	Kalendar et al., 2020	10.3390/ijms21082931
	Poaceae	*Oryza sativa indica*	Supplemental material from reference	Gao et al., 2016	10.1186/s13059-015-0867-y
	Poaceae	*Oryza sativa japonica*	Supplemental material from reference	Gao et al., 2016	10.1186/s13059-015-0867-y
	Poaceae	*Panicum virgatum*	KM262797.1	Kalendar et al., 2020	10.3390/ijms21082931
	Poaceae	*Peridictyon sanctum*	AY603376.1	Kalendar et al., 2008	10.1073/pnas.0709698105
	Poaceae	*Phleum pratense*	AF538613.1	unpublished	
	Poaceae	*Psathyrostachys fragilis*	AY271962.2	Kalendar et al., 2008	10.1073/pnas.0709698105
	Poaceae	*Saccharum hybrid*	KC686839.1	Kalendar et al., 2020	10.3390/ijms21082931
	Poaceae	*Secale cereale*	AY359471.1	Kalendar et al., 2008	10.1073/pnas.0709698105
	Poaceae	*Setaria italica*	Supplemental material from reference	Gao et al., 2016	10.1186/s13059-015-0867-y
	Poaceae	*Spartina alterniflora*	AY603377.1	Kalendar et al., 2008	10.1073/pnas.0709698105
	Poaceae	*Sorghum bicolor*	Supplemental material from reference	Gao et al., 2016	10.1186/s13059-015-0867-y

plant order	plant family	species	accession number	reference	DOI
	Poaceae	*Triticum aestivum*	AY271963.1	Kalendar et al., 2008	10.1073/pnas.0709698105
	Poaceae	*Zea mays*	AY271958.1	Kalendar et al., 2008	10.1073/pnas.0709698105
	Poaceae	*Zingeria biebersteiniana* ssp. *trichopoda*	FJ975780.1	Kalendar et al. 2020	10.3390/ijms21082931
	Poaceae	*Zingeria biebersteiniana*	FJ975777.1	Kalendar et al. 2020	10.3390/ijms21082931
	Poaceae	*Zingeria kochii*	FJ975778.1	Kalendar et al. 2020	10.3390/ijms21082931
	Poaceae	*Zingeria pisidica*	FJ975779.1	Kalendar et al. 2020	10.3390/ijms21082931
Malpighiales	Clusiaceae	*Garcinia mangostana*	EU140956.1	Kalendar et al., 2020	10.3390/ijms21082931
	Linaceae	*Linum usitatissimum*	DQ767972.1	Kalendar et al., 2008	10.1073/pnas.0709698105
	Euphorbiaceae	*Jatropha curcas*	Supplemental material from reference	Gao et al., 2016	10.1186/s13059-015-0867-y
Fabales	Fabaceae	*Cajanus cajan*	Supplemental material from reference	Gao et al., 2016	10.1186/s13059-015-0867-y
	Fabaceae	*Glycine max*	Supplemental material from reference	Gao et al., 2016	10.1186/s13059-015-0867-y
	Fabaceae	*Lens culinaris*	KX889392	Rey-Banos et al., 2016,	10.1371/journal.pone.0176728
	Fabaceae	*Lotus japonicus*	Supplemental material from reference	Gao et al., 2016	10.1186/s13059-015-0867-y
	Fabaceae	*Medicago truncatula*	Supplemental material from reference	Gao et al., 2016	10.1186/s13059-015-0867-y
	Fabaceae	*Pisum sativum*	DQ788719.1	Kalendar et al., 2008	10.1073/pnas.0709698105
Rosales	Cannabaceae	*Cannabis sativa*	Supplemental material from reference	Gao et al., 2016	10.1186/s13059-015-0867-y
	Rosaceae	*Chaenomeles japonica*	AY860309.1	Kalendar et al., 2008	10.1073/pnas.0709698105
	Rosaceae	*Fragaria x ananassa*	AY860312.1	Kalendar et al., 2008	10.1073/pnas.0709698105
	Rosaceae	*Malus domestica*	AY603366.1	Kalendar et al., 2008	10.1073/pnas.0709698105
	Rosaceae	*Prunus domestica*	AY860314.1	Kalendar et al., 2008	10.1073/pnas.0709698105
	Rosaceae	*Rosa hybrid*	AY860315.1	Kalendar et al., 2008	10.1073/pnas.0709698105
	Rosaceae	*Rosa rugosa*	AY860316.1	Kalendar et al., 2008	10.1073/pnas.0709698105
	Rosaceae	*Rubus idaeus*	AY860317.1	Kalendar et al., 2008	10.1073/pnas.0709698105
Brassicales	Brassicaceae	*Arabidopsis lyrata*	Supplemental material from reference	Gao et al., 2016	10.1186/s13059-015-0867-y
	Brassicaceae	*Arabidopsis thaliana*	Supplemental material from reference	Gao et al., 2016	10.1186/s13059-015-0867-y
	Brassicaceae	*Brassica oleracea*	AY860307.1	Kalendar et al., 2008	10.1073/pnas.0709698105
	Brassicaceae	*Brassica rapa*	AY860308.1	Kalendar et al., 2008	10.1073/pnas.0709698105
	Brassicaceae	*Thellungiella parvula*	Supplemental material from reference	Gao et al., 2016	10.1186/s13059-015-0867-y
	Brassicaceae	*Thellungiella salsuginea*	Supplemental material from reference	Gao et al., 2016	10.1186/s13059-015-0867-y

**plant order**	**plant family**	**species**	**accession number**	**reference**	**DOI**
Caryophyllales	Aioaceae	*Mesembryanthemum crystallinum*	AY603370.1	Kalendar et al., 2008	10.1073/pnas.0709698105
	Amaranthaceae	*Amaranthus palmeri*	MT230479.1	Kalendar et al., 2020	10.3390/ijms21082931
	Amaranthaceae	*Beta vulgaris*	MK216566.1	Maiwald et al., 2021	10.1093/aob/mcaa176
	Amaranthaceae	*Chenopodium quinoa*	–	Maiwald et al., 2021	10.1093/aob/mcaa176
	Caryophyllaceae	*Colobanhus quitensis*	EU882730.1	Kalendar et al., 2020	10.3390/ijms21082931
	Caryophyllaceae	*Silene latifolia*	KC686837.1	Kalendar et al., 2020	10.3390/ijms21082931
Ericales	Ericaceae	*Vaccinium corymbosum*	DQ673669.1	Kalendar et al., 2008	10.1073/pnas.0709698105

Suppl. table 1B: Data sources for published Asteraceae genomes

plant family	lineage	species	accession number	platform	reference	DOI
Asteraceae	Asteroideae	*Artemisia annua*	GCA_003112345.1	ENA	Shen *et al* ., 2018	10.1016/j.molp.2018.03.015
		Bidens hawaiensis	GCA_021521975.1	ENA	Bellinger *et al* ., 2022	10.1093/jhered/esab077
		Chrysanthemum indicum	GWHBHNH00000000	genome warehouse	–	
		Conyza (Erigeron) canadensis	GCA_000775935.1	ENA	Peng *et al* ., 2014	10.1104/pp.114.247668
		Glebionis cornaria	GCA_025667525.1	ENA	Wang *et al* ., 2022	10.1093/dnares/dsac036
		Helianthus anuus	GCA_002127325.2	ENA	Badouin *et al* ., 2017	10.1038/nature22380
		Helichrysum umbraculigerum	GCA_949592205.1	ENA	Berman *et al* ., 2023	10.1038/s41477-023-01402-3
		Mikania micrantha	GCA_009363875.1	ENA	Liu *et al* ., 2020	10.1038/s41467-019-13926-4
		Pluchea indica	GWHBCJV0000000	genome warehouse	He *et al* ., 2022	10.1038/s41559-022-01744-9
		Scalesia atractyloides	https://doi.org/10.5061/dryad.8gtht76rh	dryad	Cerca *et al* ., 2022	10.1038/s41467-022-31280-w
		Smallanthus sonchifolius	GCA_023525975.1	ENA	Fan *et al* ., 2022	10.1111/1755-0998.13675
		Stevia rebaudiana	GCA_009936405	ENA	Xu *et al* ., 2021	10.1038/s41438-021-00565-4
		Tanacetum cinerariifolium	BKCJ000000000.1	ENA	Yamashiro *et al* ., 2019	10.1038/s41598-019-54815-6
	Cichorioideae	*Cichorium endivia*	GCA_023376185.1	ENA	Fan *et al* ., 2022	10.1111/1755-0998.13675
		Cichorium intybus	GCA_023525715.1	ENA	Fan *et al* ., 2022	10.1111/1755-0998.13675
		Lactuca sativa	GCF_002870075.4	ENA	Reyes-Chin-Wo *et al* ., 2017	10.1038/ncomms14953
		Taraxakum kok-saghyz	GWHBCHF00000000	genome warehouse	Lin *et al* ., 2021	10.1007/s11427-021-2033-2
		Taraxacum mongolicum	GWHBCHG00000000	genome warehouse	Lin *et al* ., 2021	10.1007/s11427-021-2033-2
	Carduoideae	*Arctium lappa*	GCA_023525745.1	ENA	Fan *et al* ., 2022	10.1111/1755-0998.13675
		Carthamus tinctorius	GCA_001633085.1	ENA	Wu *et al* ., 2021	10.1111/pbi.13586
		Cynara cardunculus	GCA_001531365.2	ENA	Scaglione *et al* ., 2016	10.1038/srep19427

Suppl. table 1C: Data sources for published 5S rRNA genes

species	accession number	repository	reference	DOI
Ambylopyrum muticum	EU924818	NCBI	Baum et al., 2009	10.1139/g03-146
Arabidopsis lyrata	E02158	5S rRNAdb	Szymanski et al., 2016	10.1093/nar/gkv1081
Arabidopsis thaliana	E00006	5S rRNAdb	Szymanski et al., 2016	10.1093/nar/gkv1081
Avena sativa	EF071696	NCBI	Peng et al., 2008	10.1104/pp.114.247668
Beta vulgaris	Z25804	NCBI	Schmidt et al., 1994	10.1007/BF01253964
Brachypodium distachyon	XR_002960580	NCBI	Thibaud-Nissen et al., 2016	10.2527/jas2016.94supplement4184x
Brassica rapa	E02489	5S rRNAdb	Szymanski et al., 2016	10.1093/nar/gkv1081
Cannabis sativa	XR_004008092	NCBI	Thibaud-Nissen et al., 2016	10.2527/jas2016.94supplement4184x
Cajanus cajan	XR_003803880	NCBI	Thibaud-Nissen et al., 2016	10.2527/jas2016.94supplement4184x
Chrysanthemum indicum	OK181863	NCBI	-	
Cynara cardunculus	XR_003070488	NCBI	Thibaud-Nissen et al., 2016	10.2527/jas2016.94supplement4184x
Eremopyrum distans	KC188473	NCBI	Baum et al., 2013	10.1139/gen-2012-0195
Fragaria x ananassa	E00852	5S rRNAdb	Szymanski et al., 2016	10.1093/nar/gkv1081
Glycine max	XR_005890139	NCBI	Thibaud-Nissen et al., 2016	10.2527/jas2016.94supplement4184x
Henrardia persica	KC188485	NCBI	Baum et al., 2013	10.1139/gen-2012-0195
Hordeum brachyantherum	AY034775	NCBI	Baum & Johnson, 2011	10.1139/b02-057
Hordeum marinum	AF027583	NCBI	Baum & Johnson, 1998	PMID: 9809436
Hordeum vulgare	HVU07378	NCBI	Baum & Johnson, 1994	10.1139/g94-140
Jatropha curcas	E00420	5S rRNAdb	Szymanski et al., 2016	10.1093/nar/gkv1081
Lactuca sativa	E00211	5S rRNAdb	Szymanski et al., 2016	10.1093/nar/gkv1081
Linum usitatissimum	X01531	NCBI	Goldsbrough et al., 1982	10.3390/ijms22031302
Lotus japonicus	AY040715	NCBI	Pedrosa et al., 2002	10.1093/genetics/161.4.1661
Malus domestica	XR_003771672	NCBI	Thibaud-Nissen et al., 2016	10.2527/jas2016.94supplement4184x
Medicago truncatula	XR_005644521	NCBI	Thibaud-Nissen et al., 2016	10.2527/jas2016.94supplement4184x
Oryza brachyantha	XR_005812828	NCBI	Thibaud-Nissen et al., 2016	10.2527/jas2016.94supplement4184x
Oryza sativa	E00234	5S rRNAdb	Szymanski et al., 2016	10.1093/nar/gkv1081
Panicum virgatum	XR_005680461	NCBI	Thibaud-Nissen et al., 2016	10.2527/jas2016.94supplement4184x
Pisum sativum	AY499178	NCBI	Ellis et al., 1988	10.1007/BF00337732
Pyrus breitschneiderii	E02495	5S rRNAdb	Szymanski et al., 2016	10.1093/nar/gkv1081
Secale cereale	AJ307365	NCBI	Fulnecek et al., 2002	10.1007/s00438-002-0761-7
Setaria italica	KC525411	NCBI	Zhao et al., 2013	10.1186/1471-2164-14-244
Silene latifolia	AB027248	NCBI	-	
Triticum aestivum	E00299	5S rRNAdb	Szymanski et al., 2016	10.1093/nar/gkv1081
Zea mays	E00011	5S rRNAdb	Szymanski et al., 2016	10.1093/nar/gkv1081

Suppl. table 1D: Data sources for used plant read data

data for Asteraceae species

species	read accession	platform	library layout	library strategy	library source	read length
Arctium lappa	ERR5554584	illumina HiSeq 2500	paired	WGS	genomic	101
Artemisia annua	ERR11535563	illumina NovaSeq 6000	paired	WGS	genomic	150
Bidens hawaiensis	SRR14191093	pacBIO SMRT	single	WGS	genomic	-
Carthamus tinctorius	SRR2154065	illumina HiSeq 1500	paired	WGS	genomic	101
Chrysanthemum indicum	CRR389876 (genome warehouse)	illumina NovaSeq 6000	paired	WGS	genomic	150
Conyza canadensis	NA	NA	NA	NA	NA	NA
Glebionis coronaria	SRR20302831	illumina NovaSeq 6000	paired	WGS	genomic	150
Helianthus annuus	SRR2919251	illumina HiSeq 2000	paired	WGS	genomic	100
Helichrysum umbraculigerum	ERR10735438	pacBIO SMRT	single	WGS	genomic	-
Mikania micrantha	SRR8835137	illumina HiSeq X Ten	paired	WGS	genomic	150
Pluchea indica	SRR18449574	illumina NovaSeq 6000	paired	RNA-Seq	transcriptomic	150
Scalesia atractyloides	ERR9715097	pacBIO SMRT	single	WGS	genomic	-
Smallanthus sonchifolius	SRR18215734	illumina NovaSeq 6000	paired	WGS	genomic	150
Stevia rebaudiana	SRR6792730	illumina HiSeq X Ten	paired	WGS	genomic	151
Tagetes patula	SRR19579335	illumina HiSeq X Ten	paired	WGS	genomic	150
Tanacetum cinerariifolium	SRR17714824	illumina NovaSeq 6000	paired	WGS	genomic	151

data for outgroups

species	read accession	platform	library layout	library strategy	library source	read length
Beta vulgaris	SRR868931	Roche 454 GS FLX Titanium	paired	WGA	genomic	100
Fragaria x ananassa	SRR8358385	illumina HiSeq 4000	paired	WXS	genomic	150
Lotus japonicus	DRR014730	illumina HiSeq 2000	paired	WGS	genomic	100
Hordeum vulgare	SRR1804518	illumina HiSeq 2000	paired	WGS	genomic	90

Suppl. table 1E: Data sources and metadata for linkage vizualisation

data for Asteraceae species

species	genome_size* [Gbp]	genome_size_ref	DOI	read_accession	cov_used	seq_technique
Arctium lappa	1.79	Song et al., 2023	0.1186/s12870-023-04092-3	ERR5554584	0.5x	illumina
Artemisia annua	1.74	Shen et al., 2018	10.1016/j.jplph.2018.11.007	ERR11535563	1x	illumina
Bidens hawaiensis	7.56	Bellinger et al, 2022	10.1093/jhered/esab077	SRR14191093	1x	PacBio HiFi
Carthamus tinctorius	1.32	Nasab et al., 2023	10.1038/s41598-023-33347-0	SRR2154065	1x	illumina
Chrysanthemum indicum	3.02	Li et al., 2012	10.1111/j.1759-6831.2012.00241.x	CRR389876	1x	ONT
Glebionis coronaria	6.80	Wang et al., 2022	10.1093/dnares/dsac036	SRR20302831	1x	illumina
Helianthus annuus	3.60	Staton et al., 2012	10.1111/j.1365-313X.2012.05072.x	SRR2919251	0.3x	illumina
Helichrysum umbraculigerum	1.30	CATIUR000000000.1	NA	ERR10735438	1x	illumina
Mikania micrantha	1.87	Liu et al., 2020	10.1186/s12864-019-6361-7	SRR8835137	1x	illumina
Scalesia atractyloides	3.2	Cerca, 2022	10.5061/dryad.8gtht76rh	ERR9715097	1x	PacBio HiFi
Smallanthus sonchifolius	2.72	Fan et al., 2022	10.1111/1755-0998.13675	SRR18215734	1x	illumina
Stevia rebaudiana	0.40	O'Neill and Pirro, 2020	10.12688/f1000research.24396.1	SRR6792730	1x	illumina
Tagetes patula	0.43	JAOYNQ000000000.1	NA	SRR19579335	1x	illumina
Tanacetum cinerariifolium	7.10	Yamashiro et al 2019	10.1038/s41598-019-54815-6	SRR17714824	1x	illumina

data for outgroups

species	genome_size* [Gbp]	genome_size_ref	DOI	read_accession	cov_used	seq_technique
Beta vulgaris	0.75	Dohm et al., 2014	10.1038/nature12817	SRR952964	1x	illumina
Fragaria x ananassa	0.70	Isobe et al., 2018	0.1007/978-3-319-76020-9_10	SRR16002690	1x	illumina
Lotus japonicus	0.50	Mun et al., 2016	10.1038/srep39447	DRR014730	1x	illumina
Hordeum vulgare	5.10	Hisano et al., 2016	10.1186/s12864-016-3159-3	SRR21763618	1x	illumina

**** measured or assembled genome size as used for coverage calculation***
