# Supplementary material for: Evolving Together: Cassandra Retrotransposons Gradually Mirror Promoter Mutations of the 5S rRNA Genes
Source: Mol Biol Evol. 2024 Jan 23;41(2):msae010. doi: 10.1093/molbev/msae010 (PMC10853983; doi:10.1093/molbev/msae010)
Supplement: msae010_Supplementary_Data [file msae010_supplementary_data.zip › Suppl._table2_PLANT_Cassandra_metadata.pdf]

Suppl. table 2A: Properties of all Cassandra retrotransposons within the dataset. Lengths are given in bp and identical sites in LTR regions in %. Lengths are colored from lowest (yellow) to highest (green). Sequence motifs for primer binding site (PBS) and polypurin tract (PPT) are provided in the last column.

order	plant family	species	length [bp]	5' LTR [bp]	3' LTR [bp]	internal region [bp]	LTR identical sites [%]	PBS	PPT
Cyatheales	Cyatheaceae	*Sphaeropteris cooperi*	606	208	205	193	100.0	TGGTATCAGAGC	GGGGCGGTTGT
Polypodiales	Didymochlaenaceae	*Didymochlaena truncudata*	606	208	208	190	100.0	TGGTATCAGAGC	AAGGGGGCGAT
	Nephrolepidaceae	*Nephrolepis exaltata*	565	187	187	191	100.0	TGGTATCAAAGC	AAGGGGGCGAT
Piperales	Aristolochiaceae	*Saruma henryi*	563	234	234	95	100.0	TGGTATCAGAGC	GAGGGGGTGAT
Poales	Poaceae	*Ambylopyrum muticum*	724	264	264	196	100.0	TGGTATGAGAGC	GTGGGGGTGTA
	Poaceae	*Avena sativa*	773	287	286	200	99.7	TGGTATCAGAGC	GGGTGGGCGTA
	Poaceae	*Brachypodium distachyon*	668	251	251	166	100.0	TGGTATCAGAGC	GGAGGGGTGTG
	Poaceae	*Bromus sterilis*	689	266	266	157	100.0	TGGTATCAGAGC	GTGGGGGTGTA
	Poaceae	*Colpodium drakensbergense*	731	283	283	165	100.0	TGGTATCAGAGC	GGGGGGAAGGA
	Poaceae	*Colpodium versicolor*	729	282	282	165	100.0	TGGTATCAGAGC	GGGGGGAAGGA
	Poaceae	*Deschampsia antarctica*	766	283	283	200	100.0	TGGTATCAGAGC	GGGGGGGTGTG
	Poaceae	*Eremopyrum distans*	724	264	264	196	100.0	TGGTATCAGAGC	GTGGGGGTGTA
	Poaceae	*Henrardia persica*	726	264	264	198	100.0	TGGTATCAGAGC	GTGGGGGTGTA
	Poaceae	*Hordeum brachyantherum*	734	268	268	198	100.0	TGGTATCAGAGC	GTGGGGGTGTA
	Poaceae	*Hordeum marinum*	725	264	264	197	100.0	TGGTATCAGAGC	GTGGTGGTGTA
	Poaceae	*Hordeum vulgare*	725	264	264	197	100.0	TGGTATCAGAGC	GTGGTGGTGTA
	Poaceae	*Oryza brachyantha*	752	276	275	201	92.1	TGGTATCAGAGC	GGGTGGGTGTA
	Poaceae	*Oryza glaberrima*	768	281	281	206	100.0	TGGTATCAGAGC	GGGGGTGAGGG
	Poaceae	*Oryza minuta*	765	281	281	203	100.0	TGGTATCAGAGC	GGGGGTGAGGA
	Poaceae	*Oryza sativa indica*	766	280	280	206	99.6	TGGTATCAGAGC	GGGGGTGAGGG
	Poaceae	*Oryza sativa japonica*	768	281	281	206	99.3	TGGTATCAGAGC	GGGGGTGAGGG
	Poaceae	*Panicum virgatum*	869	280	280	309	100.0	TGGTATCAGAGC	GGGGGTGAGCA
	Poaceae	*Peridictyon sanctum*	732	268	268	196	100.0	TGGTATCAGAGC	GTGGGGGTGTA
	Poaceae	*Phleum pratense*	734	284	284	166	98.6	TGGTATCAGAGC	GGGGGGAAGGA
	Poaceae	*Psathyrostachys fragilis*	731	268	268	195	100.0	TGGTATCAGAGC	GTGGGGGTGTA
	Poaceae	*Saccharum hybrid*	747	273	273	201	100.0	TGGTATCAGAGC	GGGGGTGAGGA
	Poaceae	*Secale cereale*	724	264	264	196	100.0	TGGTATCAGAGC	GTGGGGGTGTA
	Poaceae	*Setaria italica*	767	281	282	204	96.8	TGGTATCAGAGT	GGGGGTGAGTA
	Poaceae	*Spartina alterniflora*	813	277	277	259	100.0	TGGTATCAGAGC	GGGGGGTGGAT

order	plant family	species	length [bp]	5' LTR [bp]	3' LTR [bp]	internal region [bp]	LTR identical sites [%]	PBS	PPT
	Poaceae	*Sorghum bicolor*	742	288	288	166	89.9	TGGTATCAGAGC	GGGGGTGAGGA
	Poaceae	*Triticum aestivum*	724	264	264	196	100.0	TGGTATCAGAGC	GTGGGGGTGTA
	Poaceae	*Zea mays*	758	280	280	198	100.0	TGGTATTAGAGC	AAAGGAGGGAT
	Poaceae	*Zingeria biebersteiniana sp. trichopoda*	731	283	283	165	100.0	TGGTATCAGAGC	GGGGGGAAGGA
	Poaceae	*Zingeria biebersteiniana*	731	283	283	165	100.0	TGGTATCAGAGC	GGGGGGAAGGA
	Poaceae	*Zingeria kochii*	729	282	282	165	100.0	TGGTATCAGAGC	GGGGGGAAGGA
	Poaceae	*Zingeria pisidica*	729	282	282	165	100.0	TGGTATCAGAGC	GGGGGGAAGGA
Malpighiales	Clusiaceae	*Garcinia mangostana*	949	439	439	71	100.0	TGGTATCAGAGC	GCTGGTGGGCA
	Euphorbiaceae	*Jatropha curcas*	830	377	379	74	89.2	TGGTATCAGAGC	GCTAGTGGGCC
	Linaceae	*Linum usitatissimum*	632	276	271	85	100.0	TGGTATCCGAGC	GGGGGGTGTAA
Fabales	Fabaceae	*Cajanus cajan*	922	434	415	73	79.5	TGGTATCAGAGC	GTTGGTGGGCT
	Fabaceae	*Glycine max*	968	444	452	72	79.6	TGGTATCATAAC	GCTGGTGGGCA
	Fabaceae	*Lens culinaris*	912	416	414	82	99.8	TGGTATCAGAGC	TTGGTGGGCCA
	Fabaceae	*Lotus japonicus*	856	393	393	70	98.7	TGGTATCAGAGC	GCAGGTGGGCC
	Fabaceae	*Medicago truncatula*	858	382	406	70	87.9	TGGTATAAGAGC	GCTGGTGGACA
	Fabaceae	*Pisum sativum*	913	421	421	71	100.0	TGGTATCAGAGC	GCTGGTGGGCA
Rosales	Cannabaceae	*Cannabis sativa*	908	431	406	71	83.4	TGGTATTACAGC	AGGGAGTTGAT
	Rosaceae	*Chaenomeles japonica*	665	297	297	71	100.0	TGGTATCAGAGC	AGGGGGTGGAT
	Rosaceae	*Fragaria x ananassa*	609	267	267	75	100.0	TGGTATCAGAGC	GGGGGGTGGAT
	Rosaceae	*Malus domestica*	643	286	286	71	100.0	TGGTATCAGAGC	GGGGGGTGGAT
	Rosaceae	*Prunus domestica*	615	270	270	75	100.0	TGGTATCAGAGC	GGGGGGTGGAT
	Rosaceae	*Rosa hybrid*	669	299	299	71	100.0	TGGTATCAGAGC	GGGGGGTGGAT
	Rosaceae	*Rosa rugosa*	670	299	299	72	100.0	TGGTATCAGAGC	GGGGGGTGGAT
	Rosaceae	*Rubus idaeus*	669	300	299	70	100.0	TGGTATCAGAGC	GGGGGGTGGAT
Brassicales	Brassicaceae	*Arabidopsis lyrata*	831	361	359	111	96.7	TGGTATCAGAGC	GGGGGGTGAAT
	Brassicaceae	*Arabidopsis thaliana*	824	356	356	112	100.0	TGGTATCAGAGC	TGTGGGTGAAT
	Brassicaceae	*Brassica oleracea*	805	350	350	105	100.0	TGGTATCAGAGC	AGGGGGTGAAT
	Brassicaceae	*Brassica rapa*	803	349	349	105	100.0	TGGTATCAGAGC	AGGGGGTGAAT
	Brassicaceae	*Thellungiella parvula*	778	344	344	90	81.8	TGGT-TCGGAGC	TATGGGTGAAT
	Brassicaceae	*Thellungiella salsuginea*	778	344	344	90	81.8	TGGT-TCGGAGC	TATGGGTGAAT

order	plant family	species	length [bp]	5' LTR [bp]	3' LTR [bp]	internal region [bp]	LTR identical sites [%]	PBS	PPT
Caryophyllales	Aioaceae	*Mesembryanthemum crystallinum*	665	300	300	65	100.0	TGGTATCAGAGC	CTGGTCAGCCC
	Amaranthaceae	*Amaranthus palmeri*	659	271	271	117	100.0	TGGTATCAGAGC	GTGGGGGTGAA
	Amaranthaceae	*Beta vulgaris*	761	283	283	195	95.4	TGGTATTAGAGC	GTGGGGGTGTA
	Amaranthaceae	*Chenopodium quinoa*	791	290	292	209	88.4	TGATATCAGAGC	GTGGGGGTGTA
	Caryophyllaceae	*Colobanhus quitensis*	801	302	302	197	100.0	TGATATCAGAGC	GTGGGGGTGAT
	Caryophyllaceae	*Silene latifolia*	806	315	315	176	96.8	TGGTATCAAAGC	TGGGGGGGAAT
Ericales	Ericaceae	*Vaccinium corymbosum*	650	235	235	180	100.0	TGGTATCAGAGC	GGTGGGGAGAA
Asterales	Asteraceae	*Arctium lappa*	647	283	283	81	100.0	TGGTATCAGAGC	GGGGGGGTGTT
	Asteraceae	*Artemisia annua*	663	299	282	82	92.0	TGGTATCAGAGC	AGGGGGGTGAT
	Asteraceae	*Bidens hawaiensis*	706	270	268	168	93.0	TGGTATCAGAGC	GAGGGGGTGTT
	Asteraceae	*Carthamus tinctorius*	616	269	266	81	96.7	TGGTATCAGAGC	GGGGGGGTGTT
	Asteraceae	*Chrysanthemum indicum*	623	269	267	87	86.6	TGGTATCAGAGC	GGGGGGTGAAT
	Asteraceae	*Conyza canadensis*	594	255	250	89	82.1	TGGTATCCAAGC	TTGAGAGGGTG
	Asteraceae	*Glebionis coronaria*	630	266	266	98	95.5	TGGTATCAGAGC	GAGGGGGTGAT
	Asteraceae	*Helianthus annus*	738	328	328	82	96.0	TTGTATCAGAGC	AGGGGGGTGAT
	Asteraceae	*Helichrysum umbraculigerum*	623	263	263	97	98.5	TGGTATCAAAGC	GGGGGGTGAGT
	Asteraceae	*Mikana micrantha*	625	270	270	85	97.0	TGGTATCAGAGC	GGGGGGGTGTA
	Asteraceae	*Pluchea indica*	601	264	256	81	74.3	TGGTATCAGAGC	ACGGGGGTGTA
	Asteraceae	*Scalesia atractyloides*	741	328	331	82	92.1	TGGTATCAGAGC	AGGGGGGTGAT
	Asteraceae	*Smallanthus sonchifolius*	641	277	277	87	93.9	TGGTATCAGAGC	GGGGGGGTATT
	Asteraceae	*Stevia rebaudiana*	732	323	328	81	89.1	TGGTATCAGAGC	AAGGGGGGTGT
	Asteraceae	*Tanacetum cinerariifolium*	613	266	266	81	88.0	TGGTATCAGGGC	AAGGGGGTGAT

Suppl. table 2B: Position of 5S similarity regions in Cassandra LTR sequences. Comparison was only done for species with a corresponding 5S rRNA gene. A-Box and C-Box motifs within the Cassandra are provided and nucleotides defining the two main variants are highlighted in bold. Ranges of the similarity region are highlighted in colour from lowest (yellow) to highest (green). Start and stop of the similarity regions are defined by the corresponding nucleotid position. The (rounded) relative values for 5S rDNA similariy region position are given in %.

species	LTR length [bp]	A-Box	C-Box	5S similiarity [nt]	similiarity start	similiarity stop	range position [%]*
Amblyopyrum muticum	264	AGTTAAGCGTGC	AG**GA**TGGGTG	80	71	151	27 - 57
Avena sativa	287	GGTTAAGCGTGC	AG**GA**TGGGTG	72	80	151	29 - 53
Brachipodium distachyon	251	AGTTAAGCATGC	AG**GA**TGGGTG	96	62	157	25 - 63
Eremopyrum distans	264	AGTTAAGCGTGC	AG**GA**TGGGTG	96	66	161	25 - 61
Henrardia persica	264	AGTTAAGCGTGC	AG**GA**TGGGTG	81	63	143	24 - 54
Hordeum brachyantherum	268	AGTTAAGCGTGC	AG**GA**TGGGTG	73	73	145	27 - 54
Hordeum marinum	264	GGTTAAGCGTGC	AG**GA**TGGGTG	78	67	144	25 - 55
Hordeum vulgare	264	GGTTAAGCGTGC	AG**GA**TGGGTG	66	80	145	30 - 55
Oryza brachyantha	276	GGTTAAGCGTGT	GG**GA**TGGGTG	87	70	155	25 - 56
Oryza sativa	281	GGTTAAGCGTGC	AG**GA**TGGGTG	75	80	154	28 - 55
Panicum virgatum	280	GGTTAAGCGTGC	GG**AT**GGGTGA	86	70	155	25 -55
Setaria italica	281	GGTTAAGCGTGC	GG**GA**TTGGTG	86	70	155	25 - 55
Secale cereale	264	AGTTAAGCGTGC	AG**GA**TGGGTG	79	66	144	25 - 55
Triticum aestivum	264	AGTTAAGCGTTC	AG**GA**TGGGTG	72	73	144	28 - 55
Zea mays	280	AGTTAAGCGTGC	GG**GA**TGGGTG	74	88	161	31 - 58
Linum usitatissimum	276	TGTTAATCGCGC	TA**AA**TGGGTG	97	108	204	39 - 74
Jatropha curcas	379	GGTTAAGCGTGC	AG**GA**TGGGTG	78	188	265	50 - 70
Glycine max	444	AGTTAAGCGTGC	GA**GA**TGGGTG	87	132	218	30 - 49
Lotus japonicus	395	AGTTAAGTGTGC	AG**GA**TGGGTG	73	238	310	60 - 78
Medicago truncatula	382	AGTTAAGCGTGC	GG**GA**TGGGTG	75	217	291	57 - 76
Pisum sativum	421	AGTTAAGCGTGC	GG**GA**TGGGTG	69	256	329	61 - 78
Cannabis sativa	431	GGTTAAGCGTGC	TG**GG**TGGATG	77	194	270	45 - 63
Fragaria x ananassa	267	GGTTAAGCATGT	AG**GA**TGGGTG	84	96	179	36 - 67
Malus domesticus	286	AGTTAAGCGAGA	AT**GA**TGGGTG	104	78	181	27 - 63
Arabidopsis lyrata	359	AGTTAAGCGTGC	AG**GA**TGGGTG	86	106	193	30 - 54
Arabidopsis thaliana	356	AGTTAAGCGTGC	AG**GA**TGGGTG	80	104	182	29 - 51
Brassica rapa	349	AGTTAAGTGTGC	AG**GA**TGGGTG	72	92	163	26 - 47

species	LTR length [bp]	A-Box	C-Box	5S similiarity [nt]	similiarity start	similiarity stop	range position [%]*
Beta vulgaris	283	AGTTAAGTGTGC	GG**GA**TGGGTG	70	90	159	32 - 56
Silene latifolia	315	AGTTAAGCGTGC	AT**GA**TGGGTG	79	113	191	36 - 61
Arctium lappa	283	GGTTAAGCGTGC	AG**GA**TGGGTG	68	122	189	43 - 67
Artemisia annua	282	AGTTAAGCGTGC	GG**CC**TGGGTG	66	118	184	42 - 65
Bidens hawaiensis	270	AGTTAAGCGTGC	AG**AT**GGTTGA	68	131	198	49 - 73
Carthamus tinctorius	269	AGTTAAGCGTGC	AG**GA**TGGGTG	68	124	191	46 - 71
Chrysanthemum indicum	269	AGTTAAGCATGC	AG**CC**TGGGTG	67	132	198	49 - 74
Conyza canadensis	255	AGTTAAGCGTGT	CT**TA**TGGGTG	66	121	187	47 - 73
Glebionis coronaria	266	AGTTAAGCGTGC	GC**TT**GGGTGA	74	126	192	47 - 72
Helianthus annuus	328	AATTAAGTGTGC	AG**GA**TGGGTG	90	154	245	47 - 75
Helichrysum umbaculigerum	263	AGTTAAGCGTGC	AG**GA**TGGGTG	65	127	191	42 - 73
Mikana micrantha	270	AGTTAAGCGTGC	GG**GA**TGGGTG	91	110	200	41 - 74
Pluchea indica	264	AGTTAAGCGTGC	TG**GA**TATGTG	77	111	187	42 - 71
Scalesia atractyloides	328	AGTTAAGCGTGC	GG**AT**GGGTGA	67	175	249	53 - 76
Smallanthus sonchifolius	277	AGTTAAGCGTGC	GG**GA**TAGGTG	67	139	196	50 - 71
Stevia rebaudiana	323	AGTTAAGCGTGA	AG**GA**TGGGTG	84	159	242	49 - 75
Tanacetum cinerariifolium	266	AGTTAAGCGTGC	GG**CC**TGGGTG	71	117	187	44 - 70

**** calclated ratios are rounded***

Suppl. table 2C: Annotation of Cassandra full length sequences in Asteraceae species. For each species the number of BLAST hits and annotated full length sequences are listed. We also provide the number of full lengths with target site duplications (TSD). Asteraceae Cassandras show several variants (last colums). Alignment pairwise identitiy values (PI) are provided in %. The observed shifts of the 5S rDNA C-box promoter motif are shaded in blue, whith the darker shade representing the cannonical motif.

species	# BLAST hits	# Cassandra	# TSD	variants	PI [%]	observed C-box motifs* Cassandra	observed C-box motifs* 5S rDNA
Artemisia annua	1107	67	47	4	78.5		
Chrysanthemum indicum	1733	152	109	2	83.3		
Glebionis cornaria	5623	495	359	2	89.6		
Tanacetum cinerariifolium	678	28	11	NA	81.7		
Conyza (Erigeron) canadensis	484	4	0	NA	84.9		
Helichrysum umbraculigerum	1327	91	45	4	85.1		
Smallanthus sonchifolius	4667	168	124	NA	85.5		
Mikania micrantha	4549	213	133	2	78.9		
Stevia rebaudiana	1356	44	25	2	76.2		
Bidens hawaiiensis	> 10000	64	35	NA	81.9		
Helianthus anuus	955	14	14	NA	92.0		
Scalesia atractyloides	6261	119	73	4	82.8		
Pluchea indica	566	38	27	2	75.3		
Arctium lappa	6605	428	334	NA	96.1		
Carthamus tinctorius	6008	67	42	2	68.1		

**** observed C-Box motifs***

**AGGATGGGTG**	GG**CC**TGGGTG	GG**CT**TGGGTG	GG**CA**TGGGTG
	GG**TA**TGGGTG	AG**CT**TGGGTG	**CTTA**TGGGTG
