# Supplementary material for: Evolving Together: Cassandra Retrotransposons Gradually Mirror Promoter Mutations of the 5S rRNA Genes
Source: Mol Biol Evol. 2024 Jan 23;41(2):msae010. doi: 10.1093/molbev/msae010 (PMC10853983; doi:10.1093/molbev/msae010)

Suppl. figure 1: Cassandra LTR alignment of different plant families. Highlighting shows differences from consensus (A=red, T=green, C=yellow, G=blue). Consensus was calculated with a treshold of 25% (bases match atleast 25% of the sequences). For Fabaceae and Asteraceae Cassandras multiple Indels for certain species are observable leading to a split in different variants. Nucleotid sequence data is provided in an additional supplemental file.

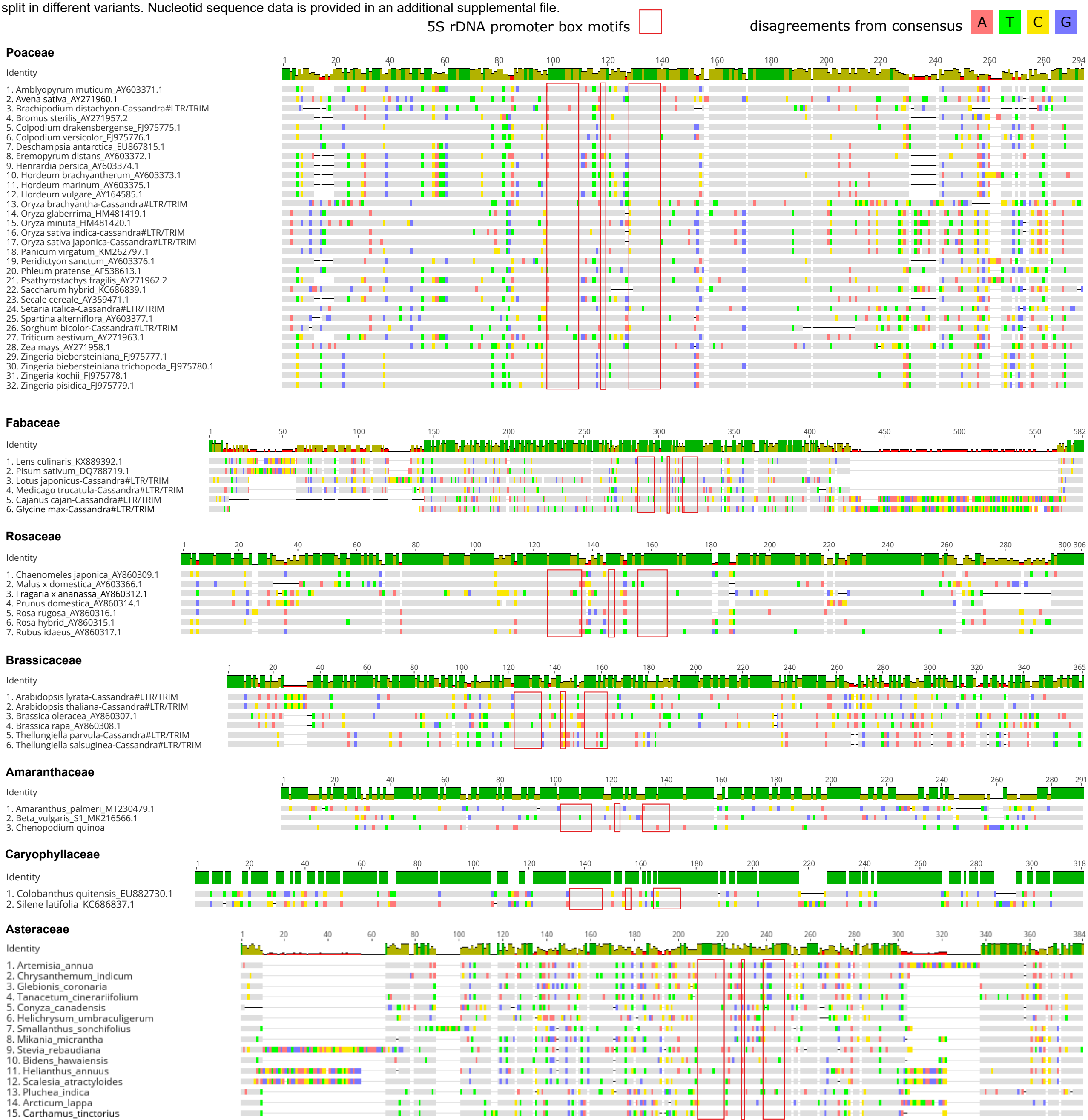

Supplement: msae010_Supplementary_Data [file msae010_supplementary_data.zip › Suppl_figure1_Cassandra_LTR_alignments.pdf]
